# A Cell-Permeable Fusion Protein Based on *Clostridium botulinum* C2 Toxin for Delivery of p53 Tumorsuppressor into Cancer Cells

**DOI:** 10.1371/journal.pone.0072455

**Published:** 2013-09-05

**Authors:** Jörg Fahrer, Johannes Rausch, Holger Barth

**Affiliations:** 1 Institute of Pharmacology and Toxicology, University of Ulm Medical Center, Ulm, Germany; 2 Institute of Toxicology, University Medical Center Mainz, Mainz, Germany; Center for Genomic Regulation, Spain

## Abstract

Genetically engineered bacterial protein toxins are attractive systems for delivery of exogenous proteins into the cytosol of mammalian cells. The binary C2 toxin from *C. botulinum* has emerged as powerful delivery vehicle, which rests on its binding/translocation component C2IIa and the genetically modified adaptor domain C2IN that act in concert to trigger cell uptake. The p53 tumor suppressor protein has a crucial function in suppressing carcinogenesis and is frequently inactivated by diverse mechanisms in human tumor cells. Therefore, we constructed a C2IN-p53 fusion protein, which is internalized into cancer cells by C2IIa. To this end, the C2IN-p53 fusion construct was overexpressed in *E. coli* with good solubility, purified by heparin affinity chromatography and protein identity was confirmed by immunoblotting. We demonstrated that the fusion protein is capable of binding to the p53 consensus-DNA with high affinity in a p53-specific manner *in vitro*. Next, the internalization of C2IN-p53 was monitored in HeLa cells by cell fractionation and immunoblot analysis, which revealed a C2IIa-mediated translocation of the fusion protein into the cytosol. The uptake was also shown in A549 and Saos-2 cells with similar efficiency. These findings were further corroborated by confocal immunofluorescence analyses of C2IN-p53/C2IIa-treated HeLa and A549 cells, displaying predominantly cytoplasmic localization of the fusion construct.

## Introduction

The C2 toxin from *Clostridium botulinum* is the prototype of binary actin-ADP-ribosylating toxins [1] and consists of the enzyme component C2I, which mono-ADP-ribosylates G-actin and the separate binding/translocation component C2II. C2II must undergo proteolytic cleavage at its N-terminus to generate the biologically active C2IIa, mediating the uptake of C2I into the host cell cytosol [1]. In solution, C2IIa forms a heptamer denoted as pre-pore and binds to its receptor found on the cell surface of all mammalian cell types tested so far [2]. After assembly with C2I, the C2I/C2IIa complex enters the cell by clathrin-mediated endocytosis in a PI3K- and Akt-dependent manner [3], [4]. In response to the acidification occurring in early endosomes, C2IIa is subjected to a conformational switch, triggering its insertion into the endosomal membrane where it forms a trans-membrane pore [5]. This allows for the translocation of C2I into the cytosol, which is promoted by host cell chaperones such as Hsp90 and peptidyl prolyl *cis/trans* isomerases [6], [7]. Subsequently, C2I catalyzes the covalent transfer of ADP-ribose onto G-actin using NAD^+^ as co-substrate [8]. This covalent modification results in a collapse of the actin cytoskeleton and finally triggers caspase-dependent cell death [9].

Owing to its properties, the binary C2 toxin has been used in numerous studies as a versatile delivery system facilitating the uptake of exogenous proteins into the host cell cytosol [10]. The N-terminal domain of C2I (C2IN) binds to C2IIa and is crucial for C2IIa-mediated internalization, but does not comprise the enzyme domain which is responsible for cytotoxic effects. Hence, the C2IN adaptor can be linked to proteins of interest by genetic means followed by expression as recombinant fusion proteins. Previously, C2IN has been fused to the virulence factor SpvB from *Salmonella enterica* to trigger its internalization into mammalian cells [11]. Another prominent example involves the bacterial C3 ADP-ribosyltransferase, a selective inhibitor of Rho GTPase, which was also expressed as C2IN fusion protein and paved the way to elucidate Rho-mediated signaling in eukaryotic cells [10], [12]. Recently, C2IN has been linked to the biotin-binding protein streptavidin, generating a versatile mammalian delivery system for biotin-labeled (macro-) molecules [13].

The tumor suppressor protein p53 is a transcription factor that is activated in response to diverse stress stimuli such as genotoxic insults and hypoxia [14]. p53 is a key player in the maintenance of genomic integrity and exerts anti-cancer activity by governing cell cycle progression and inducing apoptotic cell death in severely damaged cells. It primarily acts as a transcriptional inducer of a broad array of genes involved in cell cycle arrest and senescence, apoptosis and DNA repair [15], but is also capable of triggering apoptosis in a transcription-independent manner [16]. Of importance, p53 is found inactivated in many human tumors due to acquired mutations in its pivotal DNA binding domain [17] and increased proteolytic degradation after (poly) ubiquitination [18]. Owing to its central functions in tumor suppression, p53 is in the focus of biomedical and clinical research and numerous strategies have been developed to restore p53 in p53-deficient tumor cells [19]. One promising approach aims at the delivery of the p53 gene into tumor cells by different vehicles. It has recently been shown that polymeric microspheres loaded with chitosan-DNA nanoparticles harboring the human p53 gene are efficiently internalized into human hepatoma cells [20]. Another study reported the simultaneous uptake of the p53 gene and the antineoplastic agent doxorubicin via a hybrid nanoparticle system into HeLa cells [21]. A further interesting strategy rests on the internalization of p53 protein linked to (poly-) peptides that facilitate its uptake. To this end, p53 has been fused to gonadotropin releasing hormone (GnRH), which allows for the uptake of the fusion proteins into GnRH-positive tumor cells by receptor-mediated endocytosis [22]. Furthermore, we have shown very recently that biotin-conjugated p53 protein is internalized in a non-covalent manner by the C2-streptavidin transport system [23].

Here we report the generation of a fusion protein, in which human p53 was linked to the adaptor domain C2IN by genetic engineering, thereby facilitating its delivery by the C2IIa binding/translocation unit into cancer cells. We show that the recombinant C2IN-p53 fusion protein displays specific DNA-binding activity *in vitro* and demonstrate the C2IIa-dependent uptake of C2IN-p53 into the cytosol of diverse cancer cell lines such as HeLa cervix carcinoma and A549 lung adenocarcinoma cells.

## Results

### Genetic engineering, expression and purification of GST-tagged C2IN-p53

**Figure 1:**
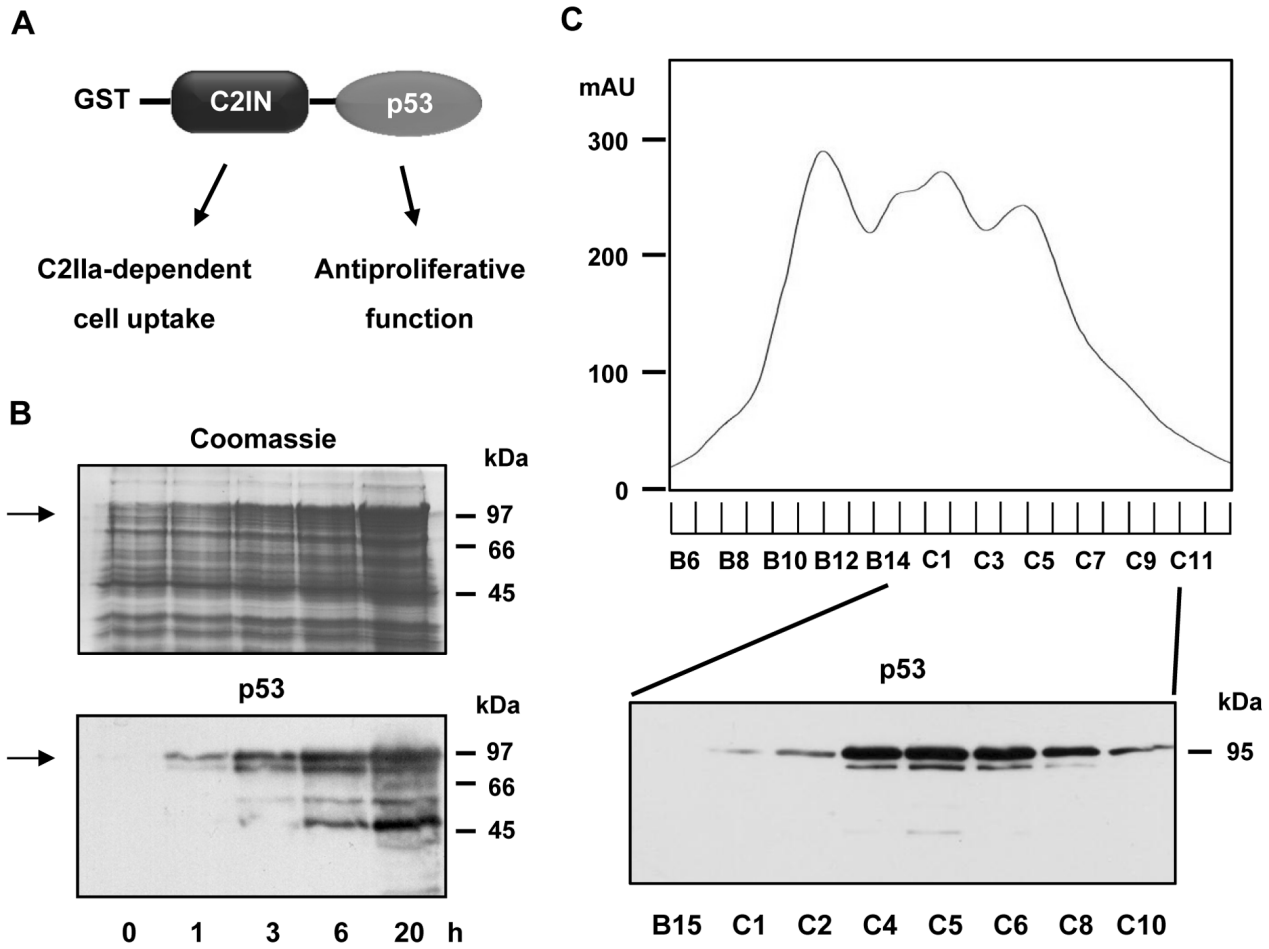
Expression and purification of recombinant GST-C2IN-p53. *A*. Scheme of GST-C2IN-p53 fusion construct. *B*. Time course of GST-C2IN-p53 expression in *E. coli* Rosetta. Samples were harvested after time points indicated (0, 1, 3, 6 and 20 h) and subjected to SDS-PAGE followed by Coomassie staining (top) or Western blot analysis using a p53 antibody (bottom). GST-C2IN-p53 is denoted by an arrow. *C*. Purification of GST-C2IN-p53 fusion protein by heparin affinity chromatography. GST-C2IN-p53 was isolated from whole cell extracts by heparin-based chromatography and eluted by a linear salt gradient as monitored by UV absorbance. Subsequently, fractions collected during affinity chromatography were characterized by immunoblot analysis with p53 antibody.

Human p53 cDNA was cloned in frame with the adaptor domain C2IN in order to generate the expression plasmid p-GEX2T-C2IN-p53, which harbors the GST-tagged C2IN-p53 fusion construct (Fig. 1A). Here, the C2IN domain mediates the C2IIa-dependent uptake of the fusion protein into the host cell cytosol, while the p53 moiety is supposed to exert antiproliferative effects upon internalization. The fusion protein was subsequently overexpressed at 16°C in *E. coli* Rosetta cells and time-dependent expression was analyzed by Coomassie staining and Western blotting (Fig. 1B). The Coomassie staining revealed a band around 97 kDa according to the expected molecular weight of GST-C2IN-p53, which increased over time. In consistency, immunoblot detection with a monoclonal antibody directed against human p53 also displayed an emerging protein band with similar molecular weight. Maximum expression of the fusion protein was found after 20 h, however with some degradations products. Expression of the construct at higher temperature (29°C) resulted in higher expression levels at the ease of considerably more degradation products (data not shown). Thus, bacterial cultures harvested after 20 h were lysed by sonication and the lysate obtained was loaded onto an anion exchange column. GST-C2IN-p53 was recovered in the flow through (data not shown) and then injected onto a heparin-sepharose column. The bound GST-C2IN-p53 was eluted by a salt gradient monitored by UV absorbance and fractions collected were assessed for p53 content by Western blotting (Fig. 1C). The GST-tagged fusion protein was detected mainly in fractions C4–C6, which corresponds to the third peak in the FPLC chromatogram. These fractions were pooled and dialyzed against low salt buffer, resulting in an average concentration of 250 ng fusion protein/ml. Of note, affinity purification of the fusion protein by glutathione-sepharose was not possible. In addition, thrombin cleavage to remove the tag was not effective, for which reason this purification step was omitted.

Taken together, a fusion construct consisting of C2IN and p53 was successfully cloned, expressed and purified by heparin affinity chromatography with sufficient yield.

### *In vitro* characterization of GST-C2IN-p53

**Figure 2:**
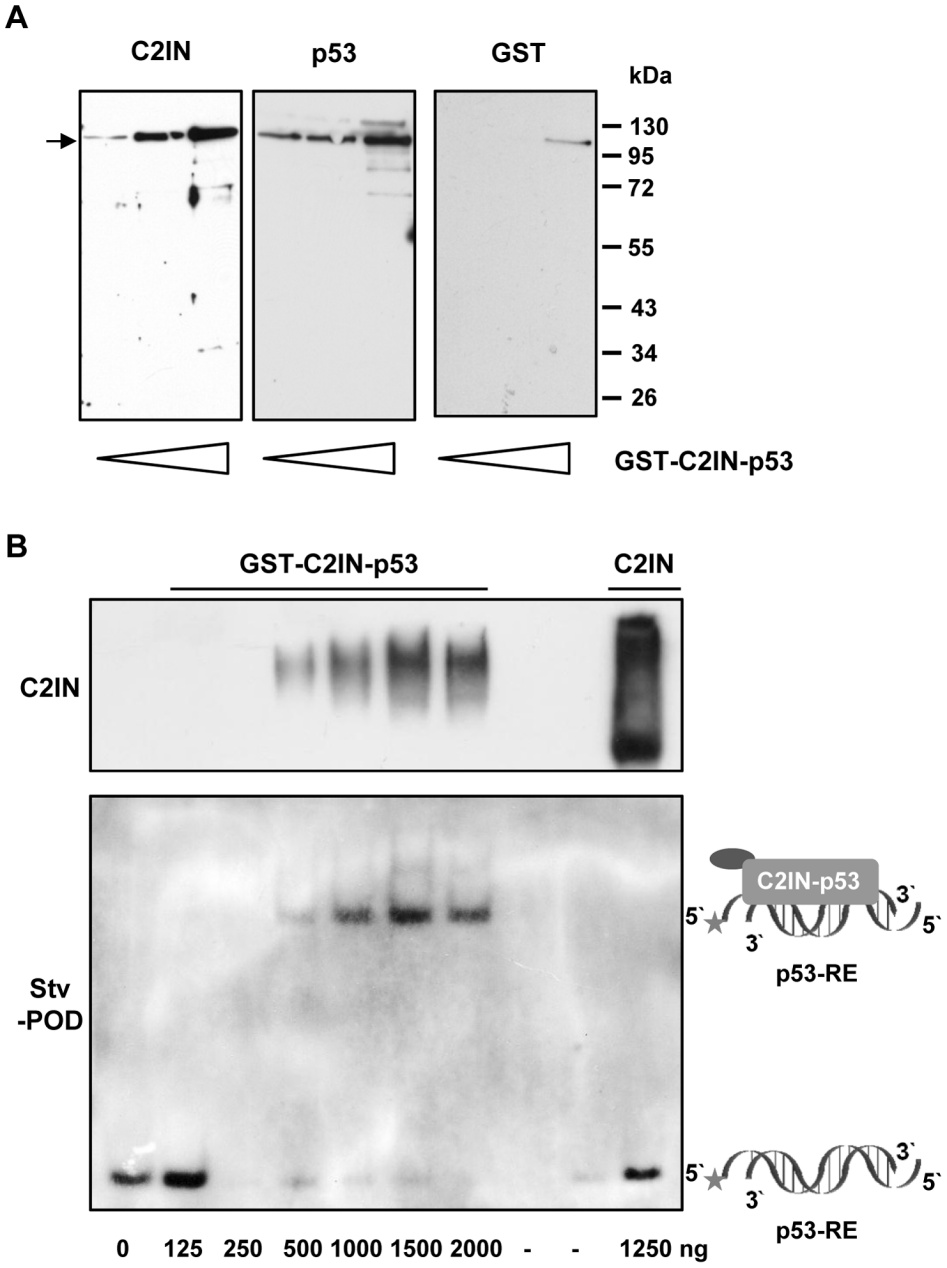
Characterization of GST-C2IN-p53 *in vitro*. *A.* Detection of different domains of the fusion protein by Western blotting. Increasing amounts of GST-C2IN-p53 (50, 100 and 200 ng) were separated by electrophoresis and subsequently analyzed by Western blotting with different antibodies (anti-C2IN, anti-p53, anti-GST). *B*. DNA-binding of purified GST-C2IN-p53. Increasing amounts of GST-C2IN-p53 were incubated with a biotin-labeled oligonucleotide duplex containing a p53-responsive element (p53-RE). Complex formation was then monitored by native PAGE followed by detection with streptavidin-POD (Stv-POD). C2IN served as negative control and was visualized by a C2IN antibody. The star denotes a biotin moiety, while the circle represents the GST-tag of the fusion protein.

The isolated recombinant GST-C2IN-p53 was first characterized by Western blotting with anti-C2IN, anti-p53 and anti-GST antibodies (Fig. 2A). Apart from the fusion protein, some minor degradation fragments were also visible, yet particularly after long exposure times. Next, the specific DNA-binding capability of GST-C2IN-p53 was determined *in vitro* by means of an electrophoretic mobility shift assay (EMSA), since this is a vital property for its transcription-dependent functions. This assay is based upon the binding of p53 to a biotinylated oligonucleotide duplex that contains the consensus sequence found in the MDM2 promotor. Incubation of this duplex with GST-C2IN-p53 resulted in the formation of high molecular weight DNA/GST-C2IN-p53 complexes in a concentration-dependent manner, with disappearance of the free oligonucleotide already at 250 ng and the occurrence of a specific DNA-protein complex at 500 ng fusion protein. Concurrently, GST-C2IN-p53 was detected by an anti-C2IN antibody. As control, recombinant C2IN (∼26 kDa) was included, which did not produce any visible complex, thereby substantiating the p53-dependent DNA-binding of the fusion protein. It should also be mentioned that the DNA binding of C2IN-p53 was similar to that of wild-type p53 (data not shown), indicating no loss of DNA-binding activity due to genetic engineering.

Collectively, the identity of GST-C2IN-p53 was confirmed by specific antibodies and the fusion protein was active *in vitro*, as it exerted specific DNA-binding activity.

### C2IIa-mediated internalization of GST-C2IN-p53 into cancer cells

**Figure 3:**
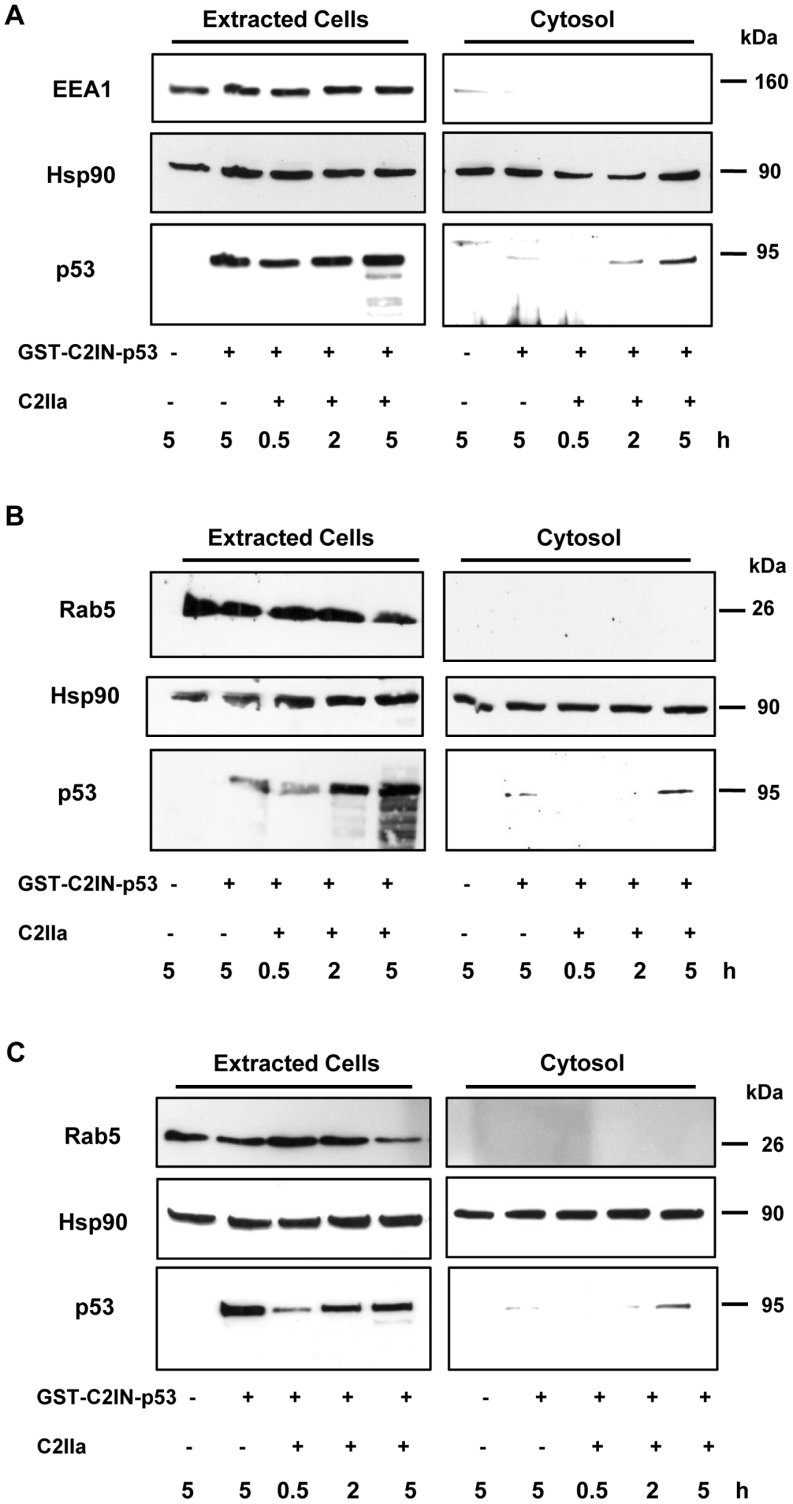
C2IIa-mediated delivery of C2IN-p53 into cancer cells. *A.* Uptake of C2IN-p53 into HeLa cervix carcinoma cells monitored by cell fractionation. HeLa cells were incubated with GST-C2IN-p53 and C2IIa for up to 5 h. Thereafter, digitonin-based cell fractionation was performed and extracted cells as well as cytosolic fractions were subjected to SDS-PAGE and subsequent Western blotting. Internalized GST-C2IN-p53 was detected by anti-p53. Hsp90 was detected as loading control, whereas EEA1 was visualized to rule out a cross-contamination of the cytosolic fraction with early endosomes. *B*. C2IIa-dependent internalization of GST-C2IN-p53 into A549 lung adenocarcinoma and Saos-2 osteosarcoma cells (*C*). Cells were treated and processed as described above, except that Rab5 was detected as marker for early endosomes.

We then addressed the question as to whether GST-C2IN-p53 is internalized into cancer cells via C2IIa. Initial experiments were performed in HeLa cells because of their strongly reduced expression of endogenous p53 [24]. HeLa cells were incubated for different times with GST-C2IN-p53 plus C2IIa and then subjected to digitonin-based cell fractionation. Immunoblot detection with p53 antibody revealed a time-dependent increase of GST-C2IN-p53 in extracted HeLa cells (Fig. 3 A, left panel), which includes cell membrane-bound and internalized protein residing in vesicles. GST-C2IN-p53 was shown to translocate into the cytosol with a maximum after 5 h (Fig. 3A, right panel), which was also detected by an anti-C2IN (data not shown). However, GST-C2IN-p53 was not detectable in the cytosol after 24 h (data not shown), which might be a consequence of its proteasomal degradation. As a negative control, cells were challenged with GST-C2IN-p53 in the absence of C2IIa. Here, GST-C2IN-p53 was detected in extracted cells, but not in the cytosol, which might be a result of non-specific binding. Early endosome antigen 1 (EEA1), a marker protein for early endosomal vesicles [25] was only found in extracted cells but not in the cytosol, demonstrating successful preparation of the cytosolic fraction without cross-contamination by early endosomes (Fig. 3A, top panel).

Prompted by these results, further cancer cell lines including A549 lung adenocarcinoma cells (p53-wt) and Saos-2 osteosarcoma cells (p53-negative) were analyzed. The C2IIa-dependent delivery of GST-C2IN-p53 into the cytosol was comparable to that observed in HeLa cells (Fig. 3B and C). It should be mentioned that some GST-C2IN-p53 was also detectable in extracted Saos-2 cells in the absence of C2IIa, which may be attributable to a non-specific binding to the cell surface and subsequent pinocytosis (Fig. 3C). Only negligible amounts of the fusion protein were visualized in digitonin-permeabilized A549 cells in the absence of C2IIa, indicating the specific C2IIa-dependent uptake in these cells (Fig. 3B). Please note that detection of the small GTPase Rab5, an established marker protein for early endosomes [26], was used as quality control for the cytosol preparation.

**Figure 4:**
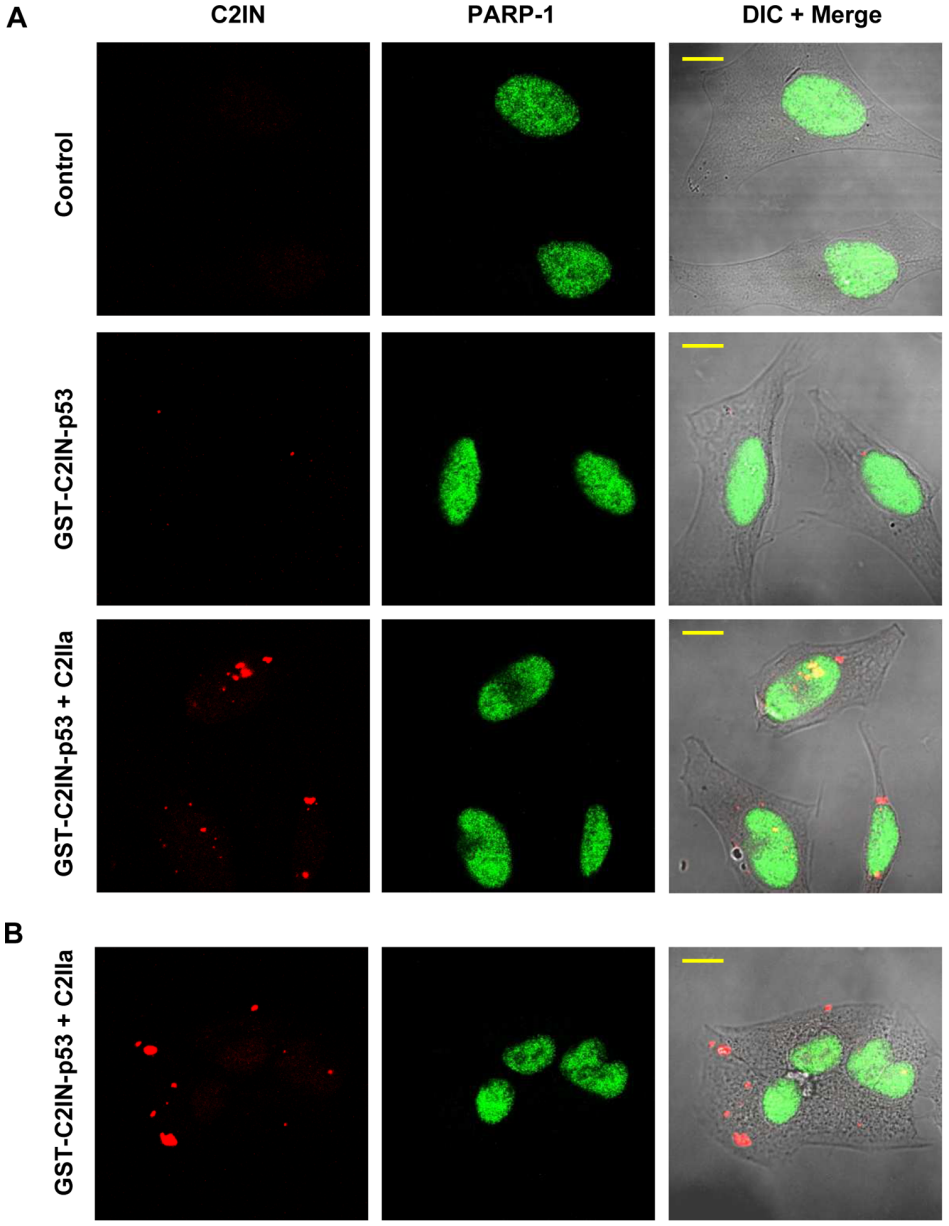
Subcellular localization of internalized GST-C2IN-p53 in HeLa and A549 cells. *A*. Confocal microscopy of GST-C2IN-p53 uptake in HeLa cells. Cells were treated with GST-C2IN-p53 and C2IIa for 5 h. As control, cells were left untreated or incubated without C2IIa. Subsequently, cells were fixed, permeabilized and stained for C2IN with a polyclonal antibody followed by an Alexa 633-coupled secondary antibody (red). PARP-1 was detected with a monoclonal antibody and subsequent staining by an Alexa 488-conjugated secondary antibody to visualize the nuclei (green). Z-Stack images were recorded in 0.75 μm optical sections and processed by ImageJ. Representative images thereof are shown. Scale bar: 10 μm. *B*. Delivery of GST-C2IN-p53 into A549 cells. Cells were treated and processed as described above. Z-Stack images were acquired in 0.75 µm optical sections and processed by ImageJ. Representative images thereof are shown. Scale bar: 10 µm.

Furthermore, the specificity of uptake and subcellular localization of GST-C2IN-p53 was analyzed in more detail by confocal immunofluorescence microscopy of HeLa cells treated for 5 h with GST-C2IN-p53 in the absence or presence of C2IIa. To visualize the nuclei, cells were counterstained for PARP-1 and 0.75 µm optical sections were acquired and evaluated for GST-C2IN-p53 using a C2IN antibody. Only very low amounts of GST-C2IN-p53 were found in the absence of C2IIa, which highlights the specificity of C2IIa-dependent uptake. The bulk of internalized GST-C2IN-p53 displayed cytoplasmic localization, but was also detected in the nucleus, where it co-localized with PARP-1 as evidenced by the yellow staining in the merged channel (Fig. 4A.). In addition, we confirmed the C2IIa-dependent delivery of GST-C2IN-p53 into A549 cells, which showed a comparable intracellular distribution as observed in HeLa cells (Fig. 4B).

In summary, we demonstrated that GST-C2IN-p53 is efficiently internalized into the cytosol of various cancer cells lines in a specific C2IIa-dependent manner.

## Discussion

The present study relies upon a genetically engineered fusion protein based on non-toxic C2 toxin which delivers the p53 tumor suppressor protein into the cytosol of various cancer cell lines. Here, p53 was cloned in frame to the adaptor domain C2IN that mediates the interaction with the C2IIa binding/translocation unit, leading to its subsequent cellular uptake. The construct was expressed as GST-tagged fusion protein and purified by heparin affinity chromatography. However, the GST-tag was shown to be deficient in glutathione binding and could not be removed by thrombin cleavage, which could be due to steric hindrance blocking the access of thrombin to its cleavage site. Yet, the remaining GST-moiety at the N-terminus of the fusion protein neither prevented the p53-mediated DNA-binding of the fusion protein *in vitro*, nor its C2IIa-dependent uptake into the cytosol of target cells. This is in line with earlier findings showing that the N-terminal GST-tag does neither inhibit the efficient C2II-mediated delivery of GST-C2I [27] nor that of GST-C2IN-C3lim fusion toxin [28] into the cytosol of HeLa cells. This is remarkable, since the correct folding of p53 is essential for its DNA-binding activity and is affected by a plethora of factors, including the redox state [29] and others [30].

An alternative strategy involves biotin-labelling of recombinant p53 and its subsequent conjugation to the C2-streptavidin transporter, as very recently demonstrated by our group [23]. However, covalent attachment of p53 to the C2IN adaptor as presented here offers a superior stability in comparison to conjugation of biotin-p53 to C2IN-streptavidin in a non-covalent manner [23], as it rests on a streptavidin moiety with decreased biotin-binding affinity [13], [31]. On the other hand this allows for intracellular release of the biotin-labeled ligand, *e.g*. by competition with endogenous biotin.

Next, we demonstrated the C2IIa-mediated uptake of the GST-C2IN-p53 fusion protein into various cancer cells lines, in which it translocated into the cytosol as confirmed by immunoblot detection upon cell fractionation. This is an interesting finding bearing in mind that the tanslocation pore formed by C2IIa in membranes of acidified endosomal vesicles is rather narrow [32] and may constrain the translocation of GST-C2IN-p53. Therefore, a partial unfolding of the fusion protein may be required, as observed for other C2IN-based fusion partners [33]. We have not analyzed the p53 functionality of the fusion protein in living cells upon internalization and, thus, cannot exclude that the translocation process with putative unfolding of C2IN-p53 may have affected its function as transcription factor, which has to be investigated in future studies. No major differences were observed regarding the cytosolic internalization among the cell lines tested. Of note, endogenous p53 was detectable neither in extracted cells nor in the cytosol of all cell lines at the settings used for Western blot analysis. This is not remarkable, since HeLa and Saos-2 cells were reported to display very low up to undetectable p53 protein levels [24], [34]. Furthermore, we have recently shown endogenous p53 in unstressed A549 cells by confocal microscopy only using high laser intensity [35] or upon induction of DNA damage by doxorubicin (data not shown). However, some unspecific binding of the fusion protein in the absence of C2IIa was revealed in extracted HeLa and Saos-2 cells. GST-C2IN-p53 accumulated in the cytosol after 5 h, but was no longer detectable after 24 h (data not shown). In consistency, a cytosolic degradation has also been reported for other C2IN-based fusion proteins, such as C2IN-C3 [36], C2IN-SpvB [37] and C2IN-streptavidin [38]. Upon internalization into HeLa and A549 cells GST-C2IN-p53 was primarily detected in the cytoplasm and less prominently in the nucleus as observed by confocal microscopy. This is in line with the notion that p53 is found in the cytoplasm in unstressed cells owing to the Crm1-dependent nuclear export in association with Mdm2 [39], [40]. However, one has to keep in mind that the subcellular localization of p53 is tightly regulated by a network of post-translational modifications and protein interactions [41], which might also be influenced by the GST-tag or the C2IN-domain.

Due to the ubiquitous expression of the C2IIa-receptor on all mammalian cells tested [42], the GST-C2IN-p53 fusion protein can be delivered into a broad range of tumor cell lines and primary cells. In order to achieve a targeted delivery, it is conceivable to modify the C2IIa binding/translocation component. To this end, the C-terminal domain of C2IIa that interacts with the cell surface receptor could be modified by genetic engineering, preserving the capability of C2IIa to translocate C2IN into the cytosol [43]. The modified C2IIa could then be fused with polypeptides such as epidermal growth factor (EGF) or somatostatin, which promote the binding to EGF- or somatastatin-receptor that are frequently overexpressed on cancer cells [44], [45]. This strategy has been described for protective antigen (PA), the binding/translocation component of the binary anthrax toxins that is closely related to C2IIa. A mutated form of PA deficient in receptor recognition was C-terminally fused to human EGF and directed the uptake of LF and EF into EGF-receptor bearing cells [46].

A potential scenario for the *in vivo* administration of C2IN-p53 in tumor therapy would be an intratumoral injection of the fusion protein in complex with native C2IIa or C2IIa modified with targeting groups as discussed above. This strategy could initially be tested in mouse xenografts obtained after injection of HeLa cells and might then be translated into the clinics for the treatment of solid tumors. However the selective delivery into tumor cells and the exclusion of putative immunogenic effects elicited by the fusion protein are crucial prerequisites for successful *in vivo* application.

In conclusion, we have constructed a fusion protein that retained its p53-dependent DNA binding activity *in vitro* and is internalized via its C2IN domain into the cytosol of various cancer cell lines using the C2IIa binding/translocation component.

## Materials and Methods

### Cloning of C2IN-p53 fusion protein

Human p53 cDNA was amplified by PCR from plasmid RcCMV-human p53, which was kindly provided by Dr. Michael Schwarz (University of Tübingen, Germany). To this end, the primers p53-I (5′-GAATTCAGATCTGAGGAGCCGCAGTC-3′) and p53-II (5′-GAATTCGGATCCTCAGTCTGAGTCAGG-3′) were used that allowed the insertion of a *Bgl*II and a *Bam*H1 restriction site. The PCR product obtained was then ligated into pCR-Blunt vector by means of the Zero Blunt PCR cloning kit (Invitrogen, Karlsruhe, Germany) and transformed into competent *E. coli* DH5á cells. The pCR-p53 plasmid was isolated and correct amplification was checked by DNA sequencing (GATC, Konstanz, Germany). Subsequently, isolated plasmid pCR-p53 was digested with *Bgl*II and *Bam*H1 to release p53 cDNA and ligated into plasmid pGEX2T-C2IN which had been linearized with *Bam*H1. After transformation in competent *E. coli* cells, correct insertion of p53 cDNA into the resulting plasmid pGEX2T-C2IN-p53 was checked by colony PCR and restriction enzyme analysis. Finally, the C2IN-p53 fusion construct was sequenced with the pGEX2T 5′- and 3′-seqencing primers (GATC, Konstanz, Germany).

### Expression and purification of recombinant C2IN-p53

The generated C2IN-p53 fusion construct was overexpressed as GST-tagged fusion protein in *E. coli* Rosetta and purified to homogeneity by affinity chromatography. To this end, *E. coli* Rosetta cells transformed with pGEX2T-C2IN-p53 were grown to an optical density of 0.6 and protein expression was induced by addition of isopropyl-ß-D-thiogalactopyranoside (IPTG). Bacterial cultures were then incubated for 20 h at 16°C and harvested by centrifugation. Cell lysis was performed by sonication in buffer containing 0.1% Triton X-100 and cellular debris was sedimented by centrifugation at 20 000 *g* for 15 min at 4°C. The clarified lysate was loaded onto a Q-sepharose Fast Flow column (GE Healthcare, Munich, Germany) and flow through containing GST-C2IN-p53 was collected. Subsequently, the sample was injected onto a heparin-sepharose column (GE Healthcare Germany) and eluted by a linear salt gradient ranging from 0 up to 1 M NaCl. Fractions with GST-C2IN-p53 were then pooled and dialyzed against a buffer consisting of 20 mM HEPES/KOH pH 7.4, 50 mM NaCl and 5 mM DTT. Protein identity of isolated GST-C2IN-p53 was confirmed by 10% SDS-PAGE and subsequent Western Blot analysis using a polyclonal C2IN antibody raised in rabbits [10], a monoclonal p53 antibody (DO-1; Santa Cruz Biotechnology, Heidelberg, Germany) and a GST antibody (BD Biosciences, Heidelberg, Germany), respectively. To analyze the time-dependent expression of GST-C2IN-p53, 100 µl aliquots of bacterial culture were collected and subjected to 10% SDS-PAGE followed by Coomassie staining and immunoblotting as described above.

### Expression and purification of C2IN and C2IIa

C2IN (∼26 kDa) and C2II (∼81 kDa) were expressed as GST-tagged proteins in *E. coli* BL21 and purified by glutathione affinity chromatography as described previously [1], [10]. After isolation of the proteins, the GST moiety was removed by thrombin-cleavage and C2II (∼61 kDa) was activated by trypsin digestion to yield C2IIa as reported [1]. Purity of the proteins was analyzed by 12.5% SDS-PAGE and subsequent Coomassie staining.

### p53 DNA binding assay

The specific DNA-binding of GST-tagged C2IN-p53 was determined by an electrophoretic mobility shift assay (EMSA). To this end, an oligonucleotide duplex harboring a p53-binding site of the MDM2 promotor was used as described previously [47]. Binding of C2IN-p53 to the oligonucleotide duplex results in high molecular weight DNA-p53 complexes with reduced electrophoretic mobility. Increasing amounts of GST-C2IN-p53 (0–2000 ng of protein) were pre-incubated in DNA-binding buffer for 10 min followed by the addition of biotin-end labeled oligonucleotide duplex (6 nM). Complex formation was allowed to occur for an additional 20 min prior to termination of the reaction by addition of EMSA loading buffer on ice. Samples were then separated by 6% (w/v) native PAGE and transferred onto a positively charged nylon membrane (GE Healthcare, Munich, Germany) by semidry blotting. Thereafter, the membrane was fixed for 60 min at 90°C and blocked with 2% (w/v) BSA in PBS-T. Free biotinylated oligonucleotide duplex and DNA-C2IN-p53 complexes were detected with streptavidin-peroxidase (1∶15,000) using enhanced chemiluminescence.

### SDS-PAGE and immunoblot analysis

Proteins were separated by SDS-PAGE and transferred to a nitrocellulose membrane (Whatman, Dassel, Germany) in a wet-blot chamber (GE Healthcare, München, Germany). The membrane was blocked with 5% (w/v) non-fat dry milk in PBS containing Tween-20 [0.1% (v/v), PBS-T] for 1 h at room temperature (RT). Subsequently, the membrane was incubated with the respective primary antibody diluted in PBS-T for 1 h at RT. After washing the membrane 3 times with PBS-T, it was probed with the appropriate secondary antibody coupled to horseradish peroxidase (Santa Cruz Biotechnology, Heidelberg, Germany) for 1 h. After further washing steps, proteins were detected by chemiluminescence using the Immobilon Western Chemiluminescent HRP Substrate (Millipore, Schwalbach, Germany).

### Cell culture

HeLa cervix carcinoma and A549 lung adenocarcinoma cells were maintained at 37°C and 5% (v/v) CO_2_ in minimal essential medium (MEM) supplemented with 10% (v/v) heat-inactivated fetal calf serum (FCS), L-glutamate (2 mM), 100 U/mL penicillin, and 100 µg/mL streptomycin. Saos-2 osteosarcoma cells were maintained in modified McCoy's 5A medium supplemented with 2 mM L-glutamine, 10% heat-inactivated FCS and antibiotics. Cells were routinely trypsinized and reseeded three times per week.

### Digitonin-based cell fractionation

Cell fractionation of HeLa, A549 or Saos-2 cells was performed essentially as described previously [13], [48]. Briefly, cells were grown in 12-well plates overnight and treated with a complex of GST-C2IN-p53 (2.5 µg/ml) and C2IIa (5 µg/ml) for the time points indicated. As a control, cells were left untreated or incubated with GST-C2IN-p53 in the absence of C2IIa. The medium was then aspirated and cells were washed 3 times with PBS. Subsequently, the cells were permeabilized by incubation with digitonin (Sigma-Aldrich, Deisenhofen, Germany) for 5 min at RT. Cells were incubated for an additional 25 min on ice to obtain the cytosolic supernatant. Afterwards, the supernatant was carefully collected and the extracted cells were scraped off. Both fractions were separated by SDS-PAGE followed by Western blot analysis. GST-C2IN-p53 was detected as stated above, using either a monoclonal p53 antibody or a polyclonal C2IN antibody. To assess equal protein loading, the cytosolic marker protein Hsp90 was detected with a monoclonal anti-Hsp90 antibody (Santa Cruz, Heidelberg, Germany). The endosomal protein EEA1 was revealed by a polyclonal antibody (Novus Biologicals, Littleton, USA) and the endosomal protein Rab5 was visualized with a monoclonal antibody (BD Biosciences, Heidelberg, Germany) to check for cross-contamination of the cytosolic fraction with early endosomal vesicles.

### Confocal microscopy

HeLa and A549 cells seeded onto cover slips were incubated with a complex consisting of GST-C2IN-p53 (2.5 µg/mL) and C2IIa (5 µg/mL). In addition, cells were either incubated only with GST-C2IN-p53 or left untreated. After incubation for 5 h, the medium was removed and cells were washed three times with PBS. Subsequently, cells were fixed with 4% (w/v) PFA, permeabilized with 0.4% (v/v) Triton X-100 and blocked with 5% (w/v) dry-milk in PBS-T. GST-C2IN-p53 was visualized with the polyclonal C2IN antibody and an appropriate Alexa647-conjugated secondary antibody (Invitrogen, Karlsruhe, Germany). Nuclei were counterstained by detection of PARP-1 using the monoclonal CII10 antibody together with an Alexa-488-coupled secondary antibody (Invitrogen, Karlsruhe, Germany). Cells were then mounted on microscope slides with Prolong Gold Antifade Solution (Invitrogen, Karlsruhe, Germany) and analyzed by confocal microscopy with a Zeiss Axiovert 200 M microscope equipped with a LSM510 Meta laser scanning device (Zeiss, Oberkochen, Germany). Z-Stack images were acquired in optical sections of 0.75 µm and processed with ImageJ (NIH, USA).

### Reproducibility of the experiments and statistics

All experiments were performed independently at least twice. Results from representative experiments are shown. Values (n≥3) are presented as means ± standard errors of the means (SEM) using GraphPad Prism4 Software.
